# Study of Arterial Blood Gas Analysis in Moderate-to-Severe COVID-19 Patients

**DOI:** 10.7759/cureus.26715

**Published:** 2022-07-10

**Authors:** Hiren Sanghani, Sumit Bansal, Vijaysinh Parmar, Rima Shah

**Affiliations:** 1 Department of Biochemistry, Shantabaa Medical College and General Hospital, Amreli, IND; 2 Anesthesiology, Max Super Speciality Hospital, Patparganj, New Delhi, IND; 3 Department of Biochemistry, Smt. Nathiba Hargovandas Lakhmichand Municipal Medical College, Ahmedabad, IND; 4 Department of Pharmacology, All India Institute of Medical Sciences (AIIMS) Rajkot, Rajkot, IND

**Keywords:** blood gas, comorbidities, respiratory alkalosis, covid-19, acid-base imbalance

## Abstract

Background

The high prevalence of pneumonia and renal involvement in coronavirus disease 2019 (COVID-19) leads to frequent acid-base abnormalities in serious patients and affects prognosis. In this study, we aimed to assess the arterial blood gas (ABG) and acid-base patterns in COVID-19 patients admitted to a tertiary care hospital.

Methodology

A retrospective observational study was conducted in a designated COVID-19 hospital involving 267 reverse transcription-polymerasechain reaction-positive COVID-19 patients. Demographic and laboratory data including ABG data within the ﬁrst day after admission and in patients with multiple ABG analyses, only the first measurement was collected and analyzed statistically, including its association with comorbidities.

Results

The most common age group of the patients was 51-60 years (30.8%), with a male predominance (male:female = 2.7:1). The most common comorbidities were hypertension, diabetes mellitus, and chronic obstructive pulmonary disease found in 147 (55%) COVID-19 patients. Alkalosis and acidosis were observed in 145 (54.3%) and 50 (18.7%) patients, respectively. The most common ABG abnormality observed was primary respiratory alkalosis with secondary metabolic acidosis in 67 (25.1%) patients, followed by primary respiratory alkalosis with secondary metabolic alkalosis in 54 (20.2%) patients. Statistically significant negative correlation was found with PaCO_2_ and pH (r = -0.530, p < 0.0001), statistically significant positive correlation was found between pH and base (r = 0.533, p < 0.0001), pH and TCO_2_ (r = 0.260, p < 0.0001), and pH and HCO_3_ (r = 0.354, p < 0.0001).

Conclusions

Acid-base abnormalities are commonly encountered in COVID-19 patients. Respiratory alkalosis as a part of a single or mixed pattern on ABG was the most common pattern found in critically ill COVID-19 patients. ABG on admission in moderate-to-severe COVID-19 patients can help in the early correction of metabolic abnormalities leading to improved patient outcomes.

## Introduction

In mid-December 2019, the novel coronavirus disease 2019 (COVID-19) epidemic was first detected in a seafood market in Wuhan, Hubei Province, China. Since then, the illness has spread to more than 215 countries/territories/areas worldwide reaching a pandemic level. The World Health Organization (WHO) designated this epidemic as a Public Health Emergency of International Concern on January 30, 2020. COVID-19 was designated a pandemic by the WHO on March 11, 2020 [1].

Patients infected with the virus show a broad variety of symptoms such as low to high-grade fever with symptoms of upper respiratory tract infection (URTI) including dry cough and sore throat. However, in some cases, the condition advances to a more severe stage marked by a dysregulated immune response and hyperinflammation, followed by the development of acute respiratory distress syndrome (ARDS). Patients might encounter various disorders such as respiratory, enteric, hepatic, and neurological conditions, including ARDS, acute cardiac injury, or secondary infection [2,3]. Patients with medical conditions such as chronic kidney disease, chronic respiratory disease, diabetes, cardiovascular disease, obesity, or cancer are more susceptible to developing severe illnesses, which may lead to death [4].

Derangement in acid-base homeostasis is common in severely ill patients. In the majority of cases, acid-base abnormalities are moderate and infrequently symptomatic and seldom have a propensity to affect organ homeostasis. On the contrary, moderate-to-severe acid-base abnormalities may lead to severe multiorgan consequences [5]. The virus’s tropism for the lungs and kidneys might result in frequent acid-base changes as a result of pneumonia and kidney impairment, respectively [6,7]. In various phases of COVID-19, numerous pathological mechanisms such as fever, inflammation involving many organs, thrombogenesis, respiratory tract infection (both lower and upper), and carotid body suppression are possible. Blood acid-base balance may move toward acidosis or alkalosis based on the underlying process [8].

The major respiratory symptom of COVID-19 is arterial hypoxia, which causes significant pulmonary mechanics abnormalities (decreased lung compliance) [9-11]. Hypoxemia caused by COVID-19 is often accompanied by an elevated alveolar-to-arterial oxygen gradient, which indicates either ventilation-perfusion mismatch or intrapulmonary shunting [12]. Arterial blood gas (ABG) analysis can help in predicting mortality among COVID-19 patients, managing the ventilatory settings for better outcomes in these patients, and can help in predicting underlying comorbid conditions in COVID-19 patients [13].

To date, different laboratory findings were detected as risk factors that can aid in disease monitoring, staging, therapy, and prognosis of COVID-19 patients. The bulk of these investigations, however, have concentrated on hematological and biochemical laboratory markers, with very little available data on ABG analysis [14].

Therefore, the present research was performed among patients with moderate-to-severe COVID-19 with the goal of determining pH, PaO_2_, PaCO_2_, and HCO_3_ (bicarbonate) levels using ABG analysis, which is indicative of acid-base abnormalities.

## Materials and methods

This was a retrospective observational study conducted from June 1, 2020, to September 28, 2020, at a designated tertiary care COVID-19 teaching hospital. The Institutional Ethics Committee (GMERS Medical College, GMERS/MCG/IEC/2021) approved the study protocol, and permission from the hospital superintendent was obtained before the initiation of the study.

All suspected COVID-19 patients who presented to the hospital during the study duration were screened. Patients aged more than 18 years, confirmed COVID-19-positive by reverse transcription-polymerase chain reaction (RT-PCR), suffering from moderate-to-severe cOVID-19 as per the WHO diagnostic guidelines, and underwent at least one ABG were included in the analysis. Patients who died before ABG analysis could be performed were not included in the study. Out of a total of 312 patients screened (512 ABG analyses), considering the inclusion and exclusion criteria, a total of 267 patients were included in the study for analysis.

All the study-related data were gathered and recorded in case record forms using files as well as reports of the patients from the medical record section. On admission, comprehensive descriptive data on weight, body mass index (BMI), gender, age, and comorbidities (medically managed diabetes mellitus type 2, hypertension, and chronic obstructive pulmonary disease (COPD)) were retrieved together with information on the day of hospitalization. In case of any query, the case details were confirmed with the treating physician. All patients were monitored by important clinical and laboratory parameters.

ABG samples were collected by trained staff using a heparinized syringe and processed immediately on a point-of-care analyzer (Abbott i-STAT analyzer, Abbott Park, IL, USA). All patients received blood gas analysis on the first day of admission, and in patients with numerous ABG analyses, we analyzed the first measurement. Arterial blood gas analysis of patients was done before any interventional management. Analysis was done in relation to anion gap, bicarbonate level, blood pH, PaCO_2_, and PaO_2_. pH levels between 7.35 and 7.45 were considered normal. For PaO_2_, values of 75 to 100 mmHg were considered normal, PaCO_2_ values of 35 to 45 mmHg were considered normal, and standard bicarbonate values (after correcting real bicarbonate value automatically in ABG) of 22 to 26 mmol/L were considered normal [15].

Epi Info software was used to evaluate the data obtained in Microsoft Excel. Actual frequencies, percentages, mean, and standard deviation are used to depict data. A logistic regression model was used for the analysis of the association between pH and PaO_2_, PaCO_2_, and HCO_3_, and the Pearson correlation coefficient was calculated. The chi-square test was used for association analysis of change in pH, PaCO_2_, PaO_2_, HCO_3_, and presence of comorbidity in patients. P-values of <0.05 were considered substantial.

## Results

The mean age of the study participants was 58.32 ± 13.4 years. Overall, 30.8% belonged to an age range of 51-60 years, followed by 61-70 years (24.7%). Out of a total of 267 patients, 195 were males and 72 were females. As shown in Table 1, the prevalence of COVID-19 patients requiring hospitalization increases progressively from 21-30 to >50 years of age, with the rise noted both in females and males. This association between the prevalence of COVID-19 patients requiring hospitalization and the age group increase was observed to be highly significant statistically (p < 0.001). Moreover, 44.9% of study patients had high BMI. Out of 267 patients, 132 had comorbidities. In total, 71 patients had hypertension, 41 patients had diabetes mellitus, six patients had COPD, 11 patients had hypertension and diabetes mellitus, two patients had hypertension and COPD, and one patient had hypertension, diabetes mellitus, and COPD (Table 1).

**Table 1:** Demographic details and comorbidities of the study patients (N = 267).

Parameter	n (%)
Age group (years)	
21–30	5 (1.9)
31–40	31 (11.6)
41–50	40 (15.0)
51–60	82 (30.7)
61–70	66 (24.7)
>70	43 (16.1)
Gender	
Males	195 (73.0)
Females	72 (27.0)
Comorbidities	
No comorbidities	135 (50.6)
Only diabetes mellitus	41 (15.4)
Only hypertension	71 (26.6)
Only chronic obstructive pulmonary disease	6 (2.2)
Hypertension + diabetes mellitus	11 (4.1)
Hypertension + chronic obstructive pulmonary disease	2 (0.7)
Hypertension + diabetes mellitus + chronic obstructive pulmonary disease	1 (0.4)
Body mass index	
Lower (<18.5)	84 (31.5)
Normal (18.5–24.9)	63 (23.6)
Higher (>25.0)	120 (44.9)

Analyzing different acid-base disorders in study patients revealed that high pH (>7.45, alkalosis) was found in 145 (54.3%) patients, low pH (<7.35, acidosis) in 50 (18.7%) patients, and normal pH in 72 (27.0%) patients. A total of 149 (55.8%) patients developed respiratory alkalosis (PaCO_2_ <35 mmHg) while 63 (23.4%) patients developed respiratory acidosis (PaCO_2_ >45 mmHg). A total of 69 (25.8%) patients had hypoxemia (PaO_2_ <75 mmHg) on ABG analysis. Elevated HCO_3_ level was seen in 41 (15.4%) patients. Table 2 shows the different parameters and their association with comorbidities. Change in pH, PaCO_2_, and PaO_2_ was significantly associated with the presence of comorbidities (p < 0.0001) while standard HCO_3_ level change was not significantly associated with comorbidities (p > 0.05).

**Table 2:** Arterial blood gas analysis and its association with comorbidities in study patients (n = 267).

Parameters	Total number of patients n (%)	Comorbidity				No Comorbidity (n = 135)	P-value
		Diabetes (n = 53)	Hypertension (n = 85)	Chronic obstructive pulmonary disease (n = 9)	Total		
pH							
<7.35	50 (18.7)	13	27	7	47	12	Chi-square = 80.1409, p < 0.0001
7.35–7.45	72 (27.0)	22	40	1	63	17	
>7.45	145 (54.3)	18	18	1	37	106	
PaCO_2_ (mmHg)							
>45	63 (23.4)	19	25	6	40	18	Chi-square = 30.1617, p < 0.0001
35–45	55 (20.1)	2	8	2	12	45	
<35	149 (55.8)	32	52	1	95	72	
PaO_2_ (mmHg)							
<75	69 (25.8)	14	28	6	46	30	Chi square = 3.3752, p = 0.1842
75–100	128 (47.9)	24	39	3	66	67	
>100	70 (26.2)	15	18	0	33	38	
Standard HCO_3_ (mmol/L)							
<22	82 (30.7)	38	37	2	77	10	Chi square = 62.6147, p < 0.0001
22–26	144 (53.9)	17	35	2	54	97	
>26	41 (15.4)	8	13	5	26	28	

As shown in Table 3, a normal ABG pattern was found in 36 (13.5%) patients on the first day of admission. Single acid-base abnormality was observed in 60 (22.4%) patients. The most common single acid-base disorder was respiratory alkalosis in 31 (11.6%), followed by metabolic alkalosis in 15 (5.6%) patients. While the most frequent abnormal ABG pattern detected was primary respiratory alkalosis with secondary metabolic acidosis in 67 (25.1%) patients, followed by primary respiratory alkalosis with secondary metabolic alkalosis in 54 (20.2%) patients.

**Table 3:** Type of arterial blood gas disorders in study patients (n = 267).

Type of acid base disorder	Frequency	%
Normal arterial blood gas	36	13.5
Metabolic acidosis	8	3.0
Metabolic alkalosis	15	5.6
Respiratory acidosis	6	2.2
Respiratory alkalosis	31	11.6
Primary respiratory alkalosis with secondary metabolic alkalosis	54	20.2
Primary respiratory alkalosis with secondary metabolic acidosis	67	25.1
Primary respiratory acidosis with secondary metabolic alkalosis	7	2.6
Primary respiratory acidosis with secondary metabolic acidosis	15	5.6
Primary metabolic alkalosis with secondary respiratory alkalosis	9	3.4
Primary metabolic alkalosis with secondary respiratory acidosis	6	2.2
Primary metabolic acidosis with secondary respiratory alkalosis	8	3.0
Primary metabolic acidosis with secondary respiratory acidosis	5	1.9

All eight patients having isolated metabolic acidosis on admission had a history of chronic kidney disease. In total, seven out of nine patients with a history of COPD presented on the day of admission with either respiratory acidosis or primary respiratory acidosis with secondary metabolic alkalosis.

**Figure 1:**
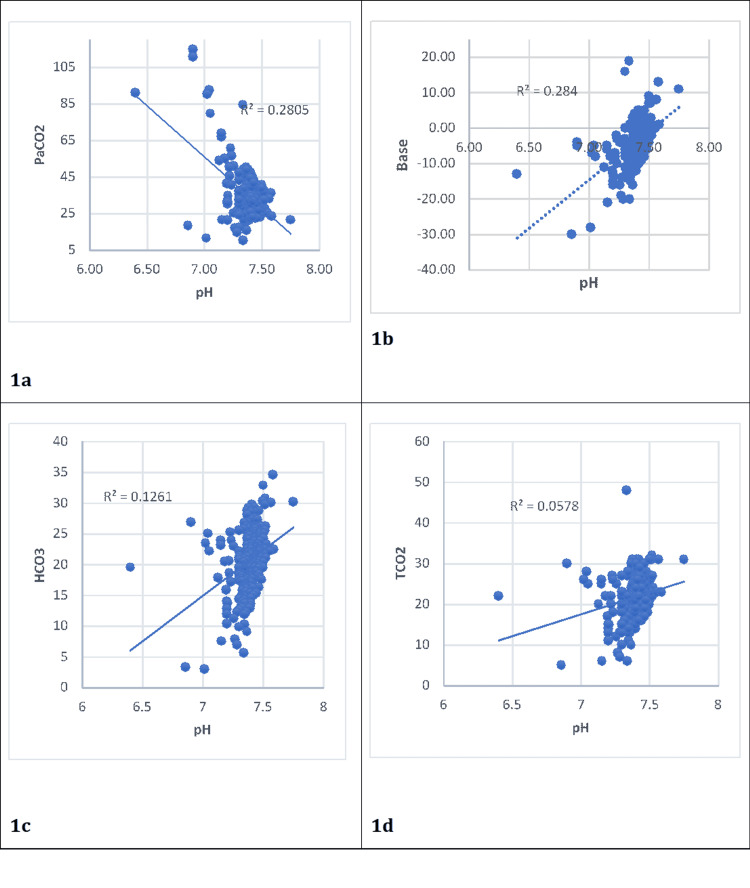
Scatter diagram of the linear association between pH and PaCO2, base, HCO3, and TCO2. (a) Scatter diagram showing the correlation between PaCO_2_ and pH. (b) Scatter diagram showing the correlation between base and pH. (c) Scatter diagram showing the correlation between HCO_3_ and pH. (d) Scatter diagram showing the correlation between TCO_2_ and pH (N = 267).

PaCO_2_ and pH (Pearson correlation coefficient (r) = -0.530, p < 0.0001) had a statistically significant negative correlation (Figure 1). A statistically significant positive correlation was examined between pH and base (r = 0.533, p < 0.0001), pH and TCO_2_ (r = 0.260, p < 0.0001), and pH and HCO_3_ (r = 0.354, p < 0.0001).

## Discussion

The COVID-19 pandemic is continuously spreading throughout the globe. With time, the virus changes its epitome and presents as a new variant with new symptoms. Pneumonia presenting as bilateral ground-glass opacities with or without high-resolution computed tomography consolidations is the primary symptom of this illness and is identified in practically all hospitalized COVID-19 patients [16]. Extensive pneumonia involving a major part of both lungs is a potentially severe infectious illness because it affects respiratory gas exchange and causes a shift in minute ventilation. Therefore, acid-base abnormality of respiratory origin was a predicted consequence in our COVID-19 patients [17]. The goal of this study was to determine arterial blood pictures, indicative of respiratory and/or metabolic alkalosis or acidosis, among moderate-to-severe COVID-19 patients [8].

Understanding the underlying pathophysiology in the study population is aided by a clear and substantial ABG pattern. The use of ABG analysis in evaluating and maintaining a patient’s oxygenation and acid-base balance is critical [13]. In intensive care unit (ICU) patients, acid-base disorders are common, with most patients suffering from metabolic acidosis, with lactic acidosis being the most frequent cause [18]. Nevertheless, COVID-19 patients were not included in previous investigations, which were conducted on general ICU patients.

Our findings showed alkalosis in approximately 54.3% of the subjects. Low levels of PaCO_2_ in 55.8% of patients show that mostly respiratory alkalosis occurs during severe COVID-19. While comparing the metabolic findings to other studies, metabolic alkalosis (33.6%) was the main modification in the study of Alfano et al. which showed a lower prevalence of metabolic alkalosis than our study [17]. The causes of respiratory acid-base problems were quickly identified. Respiratory alkalosis is caused mostly by hypoxia-induced hyperventilation, while respiratory acidosis is caused by hypercapnic respiratory failure [17].

Many theories have been proposed to explain why COVID-19 patients have respiratory alkalosis rather than acidosis but the exact mechanism is not clear. It is generally induced by a process involving hyperventilation, which includes hypoxemic causes, pulmonary diseases, and central diseases. The COVID-19-causing virus has demonstrated an affinity for angiotensin-converting enzyme 2 (ACE2) receptors, hence ACE2 receptors in the carotid body can also be a possible mechanism for this process [19].

In pulmonary disorders, hypoxic stimulation usually causes hyperventilation in an effort to repair hypoxia at the expense of CO_2_ loss [20]. Despite the fact that some patients did not exhibit severe hypoxemia in the early stages, if respiratory alkalosis occurs, they may develop compensatory hyperventilation and may rapidly deteriorate. Thus, we suggest that COVID-19 patients with respiratory alkalosis need careful monitoring, despite the fact that they have not yet shown hypoxemia [21].

The majority of patients in this study had a pH that was leaning toward alkalosis when they were admitted, and respiratory alkalosis is thought to be the outcome of viral pneumonia, which causes hypoxia without hypercapnia [22]. As the disorder progresses and the function of breathing raises, the level of PCO_2_ starts to increase, and respiratory alkalosis leads to respiratory acidosis [13].

When compared to patients whose pH was normal, our analysis demonstrated that metabolic acidosis developed in participants with changing levels of renal impairment. Patients whose pH was normal did not have this condition. It was believed that the most common reasons for metabolic acidosis in this particular population of patients included problems with ammonia excretion and a reduction in the tubular reabsorption of bicarbonate [23]. Acute or chronic metabolic acidosis is a significant medical condition that is linked to increased morbidity and a poor prognosis in patients with and without chronic kidney disease [24,25]. This acid-base condition exerts distinct detrimental consequences on the homeostasis of organs, most notably on the cardiovascular system, because acidosis decreases cardiac contractility as well as cardiac output and promotes arterial vasodilation [26-28].

We do not know why this high percentage of COVID-19 ICU patients had alkalosis, which is uncommon in critical care [29]. Some of the study patients required ventilator support and high positive end-expiratory pressure was used for them, which can be one of the contributing reason for developing respiratory alkalosis. In clinical practice, the use of corticosteroids in the past, whether at home or in any other hospital environment, may also cause metabolic alkalosis by activating the mineralocorticoid system, but it cannot be connected directly to respiratory alkalosis [8]. In this study, 15 (5.6%) patients also presented with metabolic alkalosis. Possible reasons include alkalosis caused by the kidney, with increased mineralocorticoids (endogenous or exogenic) activation as a possible source of metabolic alkalosis. The traditional RAS pathway may be activated by COVID-19, resulting in metabolic alkalosis [14]. ACE2 acts to counteract the RAS pathway’s renin and angiotensin effects (ACE2). As ACE2 is bound and degraded by severe acute respiratory syndrome coronavirus 2, its counter-regulatory actions may be reduced. It is possible that angiotensin II and aldosterone, both of which increase RAS activity, will stimulate salt reabsorption inside the distal nephron and increase the excretion of potassium in the urine [30]. The RAS is largely responsible for regulating blood pressure, as well as electrolyte concentration and water balance, along with the acid-base state of the organism. It is composed of two branches that have been thoroughly discussed, namely, the protective pathway and classic vasoconstrictive. The classic process results in the production of aldosterone, oxidative stress, fibrosis, cell proliferation, and vasoconstriction, all of which result in metabolic alkalosis [14].

Our study highlighted acid-base abnormalities in COVID-19 patients and their association with different comorbidities. These acid-base abnormalities were more prevalent in patients with either hypertension, diabetes, or COPD as comorbidities. The exact reason for such a correlation could not be identified but further research in this area can be conducted. The fact that our research was conducted at a single location and only focused on patients’ admission ABG, as well as biochemistry findings, is one of the study’s limitations. Due to the retrospective nature of the research and the lack of a control group, it is difficult to draw any conclusions about the significance of these findings. Another disadvantage is that these individuals lacked a history of either chronic drug use before hospitalization or any alternative medications they may have consumed. However, to validate acid-base abnormalities distribution in patients with COVID-19, larger investigations free from the possibility of a selection bias are needed.

## Conclusions

A significant percentage of acid-base abnormalities were found in individuals who were admitted to the hospital for symptoms related to COVID-19. They experienced numerous acid-base alterations, and such variations were more significant in patients with comorbidities. The most prevalent pattern noted in moderate to severally ill COVID-19 patients was one that showed up on their ABG analyses as respiratory alkalosis as part of a single or mixed pattern. ABG on admission in moderate-to-severe COVID-19 patients can help in the early correction of metabolic abnormalities and may lead to improved outcomes for the patients.
